# Preparation and Investigation of Cellulose Acetate/Gelatin Janus Nanofiber Wound Dressings Loaded with Zinc Oxide or Curcumin for Enhanced Antimicrobial Activity

**DOI:** 10.3390/membranes14050095

**Published:** 2024-04-23

**Authors:** Tianyue Huang, YuE Zeng, Chaofei Li, Zhengqing Zhou, Yukang Liu, Jie Xu, Lean Wang, Deng-Guang Yu, Ke Wang

**Affiliations:** 1 School of Materials and Chemistry, University of Shanghai for Science and Technology, 516 Jungong Road, Shanghai 200093, China; 213353276@st.usst.edu.cn (T.H.); 222203101@st.usst.edu.cn (Z.Z.); lk17855516270@163.com (Y.L.); 2135051523@st.usst.edu.cn (J.X.); 2135053719@st.usst.edu.cn (L.W.); 2 Department of Neurology, RuiJin Hospital Lu Wan Branch, Shanghai Jiao Tong University School of Medicine, Shanghai 200025, China; lw10317zye@rjlwh.com.cn; 3 Department of General Surgery, Ruijin Hospital Affiliated to Shanghai Jiao Tong University School of Medicine, Shanghai 200025, China; lcf11899@rjh.com.cn

**Keywords:** cellulose acetate, gelatin, zinc oxide, curcumin, electrospinning, Janus structure, antimicrobial dressings

## Abstract

The skin, as the largest organ, serves as a protective barrier against external stimuli. However, when the skin is injured, wound healing becomes a complex process influenced by physiological conditions, bacterial infections, and inflammation. To improve the process of wound healing, a variety of wound dressings with antibacterial qualities have been created. Electrospun nanofibers have gained significant attention in wound dressing research due to their large specific surface area and unique structure. One interesting method for creating Janus-structured nanofibers is side-by-side electrospinning. This work used side-by-side electrospinning to make cellulose acetate/gelatin Janus nanofibers. Curcumin and zinc oxide nanoparticles were added to these nanofibers. We studied Janus nanofibers’ physicochemical characteristics and abilities to regulate small-molecule medication release. Janus nanofibers coated with zinc oxide nanoparticles and curcumin were also tested for antibacterial activity. The Janus nanofibers with specified physicochemical characteristics were successfully fabricated. Nanofibers released small-molecule medicines in a controlled manner. Additionally, the Janus nanofibers loaded with curcumin exhibited excellent antibacterial capabilities. This research contributes to the development of advanced wound dressings for promoting wound healing and combating bacterial infections.

## 1. Introduction

The skin, being the largest organ of the body, plays a crucial role in protecting the body from external stimuli, regulating temperature, and maintaining moisture levels. However, when the skin’s integrity is compromised, it can lead to the formation of wounds. The increasing global elderly population has resulted in a growing burden on the healthcare system due to chronic wounds [1,2,3,4]. Wound healing is a complex process influenced by various factors, including the patient’s physiological condition, bacterial infections, and inflammation, all of which can impede the healing process and re-epithelialization [5]. Wounds that have not undergone complete healing and have not acquired both anatomical and functional integrity within a month are often classified as chronic [6]. Chronic wounds may arise due to several conditions, such as chronic illness, inadequate blood supply, diabetes, poor nutrition, age, and local issues including pressure, infection, and swelling [7]. This can result in persistent inflammation challenging wound healing or even tissue necrosis [8].

To prevent the formation of long-lasting wounds, a range of techniques are employed in the field of wound care, including debridement, antibiotic treatment, and the application of wound dressings [9]. Ideal wound dressings should create an optimal moisture level at the wound site, absorb fluid from the wound surface, act as a barrier against microbes, and facilitate gas exchange through a carefully designed mechanism. Moreover, these dressings should be non-toxic, non-allergenic, possess good cytocompatibility, and exhibit antibacterial properties to expedite wound healing [10].

Nanofiber wound dressings have gained significant attention due to their unique structure and ability to adapt their physical and mechanical characteristics to promote wound healing [11,12,13,14]. Nanofibers possess a large specific surface area, enabling efficient loading of drugs and controlled release. Additionally, their structure closely resembles the collagen or elastin fibers found in the extracellular matrix (ECM) of healthy skin. This similarity enables nanofibers to provide additional support for fibroblasts and keratinocytes, facilitating their attachment and migration towards the wound site, ultimately aiding tissue regeneration and wound closure [15]. 

Recent advancements in electrospun nanofiber technology have introduced novel approaches for the development of wound dressings, such as blend electrospinning materials [16,17], coaxial electrospinning [18,19], and side-by-side electrospinning [20,21]. These new techniques overcome the limitations of the traditional single-polymer, single-axis electrospinning method by allowing the simultaneous electrospinning of multiple polymers, thus maximizing the benefits of each polymer while mitigating their limitations [22,23,24,25]. Moreover, the advancement of multi-fluid electrospinning simplifies the spinning process and enables the creation of nanofibers with unique shapes [26]. Side-by-side electrospinning, in particular, enables the fabrication of nanofibers with a Janus structure, exhibiting distinct characteristics on each side. This dual-sided structure holds great potential for controlled release of pharmaceuticals and medicinal products [27,28].

Gelatin (Gel), a hydrolyzed form of collagen, is highly regarded in the medical field due to its superior biocompatibility, degradability, and hydrophilicity. However, its weak mechanical properties and rapid degradation rate restrict its use [29]. On the other hand, cellulose, a polysaccharide derived from plants, exhibits excellent biocompatibility and has long been used as a wound dressing material. However, its low solubility in many solvents limits its processability. Cellulose acetate (CA), a derivative of cellulose, possesses excellent solubility and electrospinnability [30]. CA nanofibers have shown the ability to regulate drug release and are commonly employed in electrospun drug delivery systems [31].

**Scheme 1:**
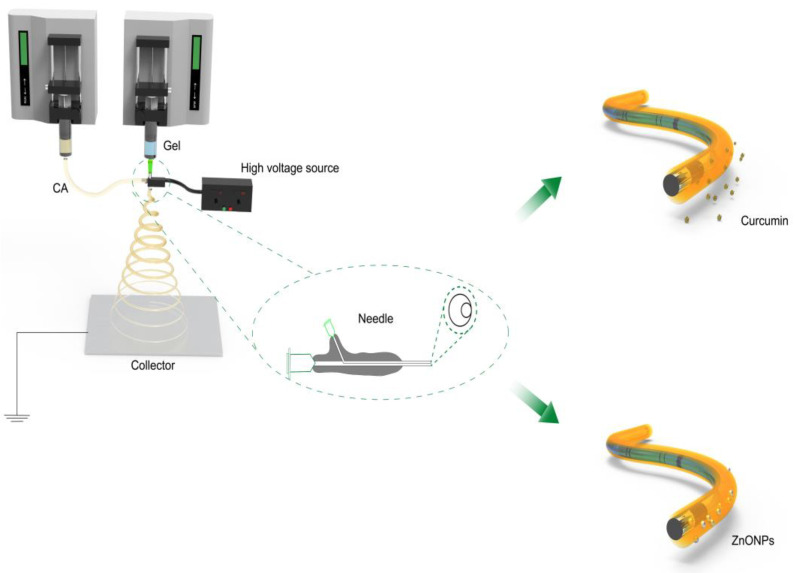
Schematic of side-by-side electrospinning device and Janus nanofiber loaded with two different drugs.

In this study, side-by-side electrospinning was employed to create Janus nanofibers by incorporating CA and Gel in a single nanofiber, with Gel as a hydrophilic polymer that adds many hydroxyl and amino groups to Janus nanofibers. Janus nanofibers’ weak CA hydrophilicity was enhanced [32,33]. This ultimately leads to improved biocompatibility of the Janus nanofibers. Meanwhile, in Janus nanofiber, the addition of CA enhances the weaker mechanical characteristics of the Gel. Two compounds, zinc oxide (ZnO) and curcumin (CUR), known for their antibacterial properties, were incorporated onto the CA nanofibers [34,35]. The study investigated the influence of incorporating nanoparticles (NPs) and small-molecule medicines into Janus nanofibers on the antibacterial properties of the nanofiber membranes (Scheme 1). Furthermore, the impact of Janus nanofibers on the release of CUR during the small-molecule drug delivery process was examined.

## 2. Materials and Methods

### 2.1. Materials

Cellulose acetate (CA), Mn~3000 (Shanghai Jiji Biochemical Technology Co., Shanghai, China) was used. Gelatin (Gel) was acquired from pig skin (Sinopharm Chemical Reagent Co., Shanghai, China). Curcumin (CUR) 98% was used (Shanghai Maclean Biotechnology Co., Shanghai, China). Zinc oxide (ZnO) nanoparticle size was 30 ± 10 nm (Shanghai Maclean Biotechnology Co.). Two different solvents, 2,2,2-trifluoroethanol (TFEA), AR and 1,1,1,3,3,3-hexafluoroisopropanol (HFIP), AR (Shanghai Maclean Biotechnology Co., Shanghai, China) were used. Phosphate buffered solution (PBS) tablets (Sinopharm Chemical Reagent Co., Shanghai, China) were used. Deionized water is made in-house by a deionized water machine.

### 2.2. Preparation of Nanofiber by Electrospinning

To prepare a 12% (*w*/*v*) solution of CA, 1.2 g of CA was added to 10 mL of HFIP. The solution was agitated for almost 8 h until the CA was fully dissolved. Then, 0.2 g of ZnO nanoparticles were put into a solution of CA and agitated for over 24 h to produce a cloudy white mixture. Prior to electrospinning, the fluid was subjected to ultrasonication for 30 min to ensure the uniform dispersion of ZnO nanoparticles. Then, 0.2 g of CUR was added to a separate batch of CA solution and stirred until completely dissolved. A spinning solution was made by dissolving 1.4 g of Gel in 10 mL of TFEA to create a 14% (*w/v*) Gel spinning solution, which was agitated until fully dissolved. 

The electrospinning setup consisted of five components: a KDS 200 propulsion pump, a high-voltage generator, a receiver unit, a 20 mL syringe, and a custom-made spinning needle. The prepared solution was transferred into a 20 mL syringe and then attached to the propulsion pump. In the uniaxial electrospinning process, the syringe was directly connected to the needle. For side-by-side electrospinning, the syringe containing the Gel solution was directly connected to the custom-made needle, while the syringe containing the CA solution was connected to the needle using a tubing piece. The solutions that include Gel are located on the circular side, whereas the solutions containing CA are located on the crescent side. The high-voltage power source was connected to the metallic part of the needle using crocodile clips. A collector was created by placing an aluminum foil-covered cardboard box, grounded, 18 cm below the needle (see Scheme 1). CA nanofibers and Gel nanofibers were fabricated using uniaxial electrospinning. A unique nanofiber with a Janus structure was created using side-by-side electrospinning utilizing a custom-made needle. The Janus nanofiber without any medications is denoted as F0, whereas the Janus nanofiber containing CUR is designated as F1, and the Janus nanofiber incorporating ZnO is labeled as F2. The electrospinning process was conducted at a temperature of 20 ± 4 °C and a relative humidity of 41 ± 5%. Table 1 presents the experimental parameters for both uniaxial electrospinning and side-by-side electrospinning.

### 2.3. Characterization of Janus Nanofiber Surface Structure

Scanning electron microscopy (SEM), (Quanta FEG450, FEI Limited from Hillsboro, FL, USA) was employed to investigate the morphology of the nanofiber films. The samples were affixed to a sample stage using conductive glue, followed by the application of a gold sputter coating for 120 s in a nitrogen environment. Subsequently, scanning electron microscope images of the nanofiber surface were captured. Energy-dispersive X-ray spectroscopy (EDS) (Quanta FEG450, FEI Limited from Hillsboro, Florida, USA) was used to conduct elemental analysis of the surface of F2 nanofibers. The diameters of the nanofibers were randomly measured using image analysis software (Image J v1.53t, Bethesda, MD, USA). The collected data were statistically analyzed to determine the average diameters and the distribution trend of the diameters.

Transmission electron microscopy (TEM) (Tecnai G2 F 30, FEI Limited from Hillsboro, FL, USA) was utilized to examine the distinct nanostructures within the nanofibers of group F2. Initially, the nanofibers were collected onto a 200-mesh lace carbon film pass-through grid. The internal structure of each fiber was then analyzed using transmission electron microscopy at 100 kV.

### 2.4. Characterization of Janus Nanofiber Constituents

The functional groups present in the nanofiber were identified using Fourier transform infrared spectroscopy (FTIR) using KBr as the background material. A small amount of nanofiber membrane sample was carefully placed into thoroughly dried KBr, and the KBr and fiber membranes were combined through pulverization. The NF samples were scanned using transmission mode. The scan spanned from 500 to 4000 cm^−1^ with a 2 cm^−1^ resolution. ZnO was subsequently examined using an X-ray diffractometer (XRD). The X-ray source utilized Cu Kα radiation (λ = 1.541 Å) with a scanning range of 10–80° and a scanning speed of 5° per minute. The X-ray generator was used at 40 kilovolts and 30 milliamperes.

### 2.5. Wetting Property Analysis

The material’s wettability was assessed utilizing the water contact angle technique. The samples were sliced into 4 cm × 4 cm dimensions and affixed to slides. Approximately 10 microliters of water was applied to the sample surface, and the water contact angle was determined using an interfacial tension meter (JC2000C1, Shanghai Zhongchen Digital Technic Apparatus Co., Shanghai, China).

### 2.6. Mechanical Properties Analysis

The nanofiber’s mechanical characteristics were evaluated using a precise microtensile tester. The nanofiber film was sliced into dumbbell-shaped sample strips of 5 mm in width using a sample fabrication machine. The thickness was measured with a spiral micrometer, and the sample strips were secured on the tensile testing equipment to conduct tensile tests at a constant rate of 1 mm/min. Tensile strength and fracture toughness were determined based on the tensile test outcomes.

### 2.7. In Vitro Drug Release Process

A sample was weighed and then plunged into 500 mL of pre-configured PBS in a covered beaker. The beaker was put on a shaker at 35 °C and 50 rpm. Periodically, 4 mL of liquid was removed from the beaker and an equal volume of PBS was added to the beaker thereafter. To account for the quick drug release at the beginning, short sample intervals were used initially and were extended later as needed. According to the Chinese Pharmacopoeia (2020), the highest UV absorption peak of CUR is at 276 nm. Initially, CUR was dissolved in PBS to create various concentrations of the standard solution, and its UV absorbance at 276 nm was measured. The absorbance and concentration were analyzed using linear fitting to establish a linear equation for the absorbance–concentration correlation. The absorbance of samples collected during the release process was measured to determine the concentration C_1_ using a linear equation. The theoretical concentration C_0_ was then calculated based on the weight of the samples. The release ratio of the drug at that time was determined by the percentage of the two concentrations. A graph was created by plotting the time against the release ratio to illustrate the medication release pattern.

### 2.8. In Vitro Antimicrobial Performance Testing

The in vitro antimicrobial investigations were conducted utilizing the patch technique, with the nanofiber being sliced into 6 mm diameter prototypes. The nanofibers were placed in the middle of the culture medium containing *Escherichia coli* and *Staphylococcus aureus*. After 24 h of incubation, the nanofiber was taken out to examine the surrounding community and measure the inhibition circle’s size to assess its antimicrobial effectiveness.

## 3. Results

### 3.1. Structure of Janus Nanofiber

**Figure 1:**
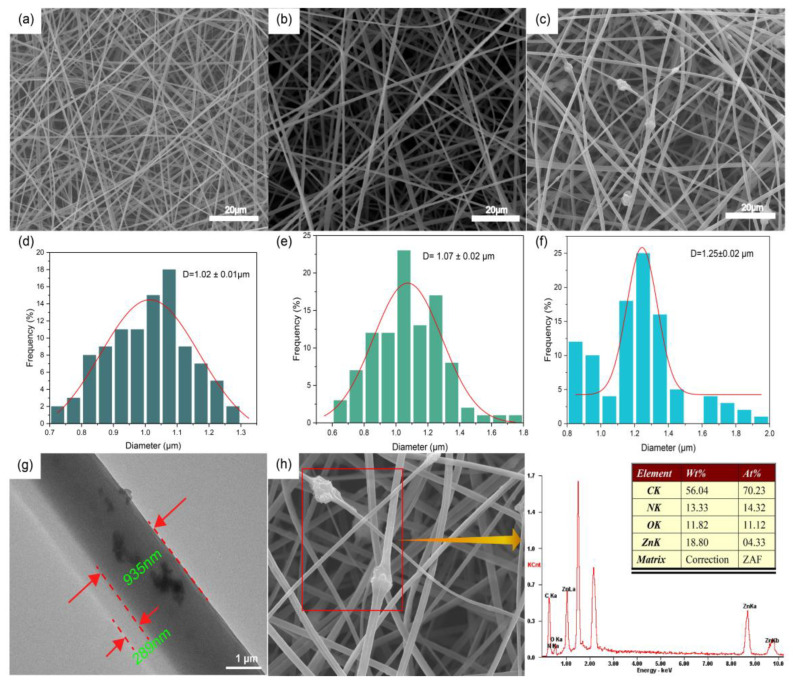
SEM images of Janus nanofiber membranes (**a**) F0; (**b**) F1; (**c**) F2. Statistical analysis of fiber diameter of (**d**) F0, (**e**) F1, (**f**) F2 and (**g**) TEM image of F2. (**h**) EDS results of F2 nanofiber surface.

The SEM images of the nanofiber surface exhibited a smooth texture without prominent beading, indicating improved compatibility between the two working fluids in the Janus nanofiber. The diameter of F0, without drug loading, was 1.02 ± 0.01 μm (Figure 1a,d), while the diameter of F2 increased to 1.25 ± 0.02 μm (Figure 1c,f). The diameter of F1 remained relatively unchanged at 1.07 ± 0.02 μm (Figure 1b,e). The presence of the medication CUR, being a small molecule, allowed it to dissolve in the working fluid without significantly affecting the nanofiber diameter. The SEM images of F2 nanofibers depict the presence of clusters that are attached to the nanofibers. These clusters were analyzed using EDS and were found to predominantly consist of ZnO (Figure 1h). Agglomeration of the ZnO nanoparticles occurred, leading to the formation of larger clusters. As a consequence, the diameter of the F2 nanofiber increased due to the presence of these clusters. Transmission electron microscopy (TEM) observations revealed a distinct Janus structure of the nanofibers, with evident particles present inside them (Figure 1g). 

### 3.2. Composition of Janus Nanofiber

**Figure 2:**
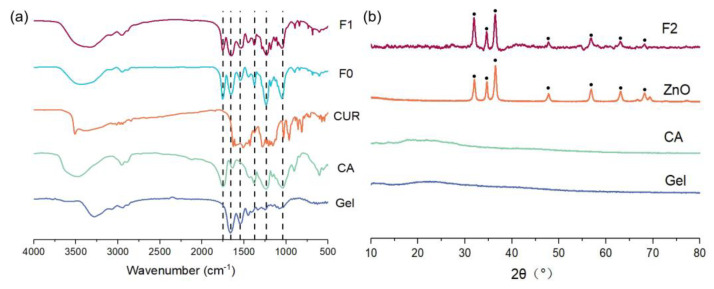
(**a**) FTIR patterns of F0, F1, CA, Gel, and CUR. (**b**) XRD patterns of CA, Gel, F2, and ZnO.

FTIR analysis was conducted on F0 and F1 (Figure 2a) to determine the composition of the Janus nanofiber membranes. The FTIR data revealed characteristic peaks at 1760 cm^−1^ and 1240 cm^−1^, corresponding to CA, in all three groups [17]. Additionally, distinctive peaks at 1650 cm^−1^ and 1540 cm^−1^, attributed to Gel, were observed [36]. The presence of specific peaks at 1290, 964, and 814 cm^−1^ confirmed the incorporation of CUR into the nanofiber [37]. These distinct peaks confirmed that the Janus nanofiber consists of a bicomponent structure comprising CA and Gel, with effective integration of CUR. To detect ZnO, which was challenging using FTIR, XRD analysis was performed specifically on the F2 group (Figure 2b). The XRD pattern of F2 exhibited diffraction peaks at 31.9°, 34.6°, 36.4°, 47.6°, and 56.7°, indicating the presence of ZnO crystal diffraction peaks and confirming the successful loading of ZnO nanoparticles onto the Janus nanofiber [38].

### 3.3. Wetting Properties of Janus Nanofibers

**Figure 3:**
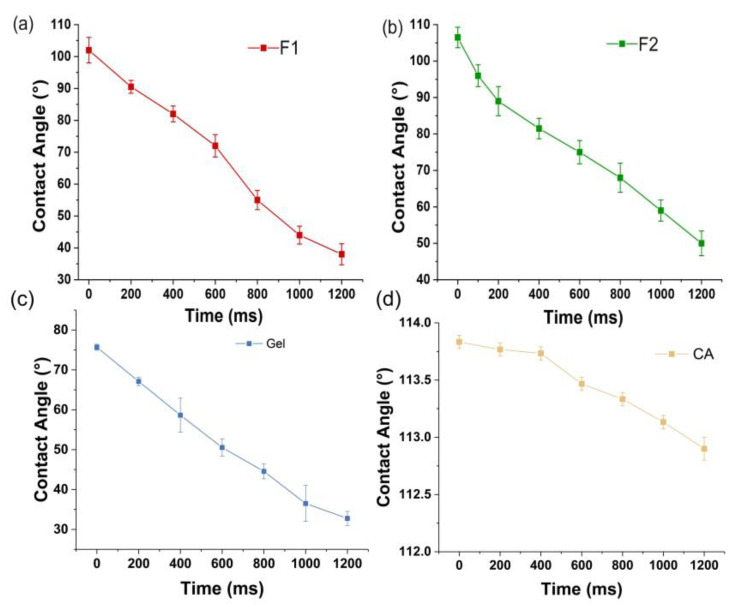
Water contact angle with time. (**a**) F1 nanofiber membrane. (**b**) F2 nanofiber membrane. (**c**) Gel nanofiber membrane. (**d**) CA nanofiber membrane.

Optimal hydrophilicity and wettability of bioactive fiber membrane dressings play a crucial role in promoting wound healing [39]. The wettability of bioactive dressings can be assessed by measuring the contact angle, which represents the angle formed by a liquid on a solid surface [40]. This angle can be directly measured using an interfacial tension meter. It is evident that the water contact angle of the Janus nanofiber decreases rapidly within a short period and can reach a minimum of 50° or less (Figure 3a,b). This indicates that the Janus material itself exhibits hydrophilicity. The use of a hydrophilic Gel (Figure 3c) enhances the limited hydrophilicity of CA (Figure 3d). Furthermore, the addition of CUR small molecules and ZnO NPs does not alter this hydrophilicity, which is beneficial for cell adhesion and proliferation at the wound site, ultimately accelerating the wound healing process [5].

### 3.4. Mechanical Properties of Janus Nanofibers

**Figure 4:**
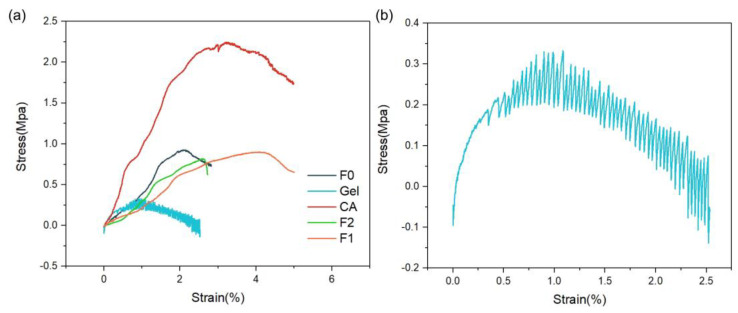
(**a**) Mechanical properties of different nanofibers. (**b**) Mechanical properties of Gel.

The mechanical properties of nanofiber membrane wound dressings are crucial for maintaining stability and integrity under external pressures and stretching, thereby protecting wounds and promoting wound healing [41,42]. Mechanical property experiments were conducted on Janus nanofiber membranes to assess their performance in this regard (Figure 4a). The results revealed that Gel exhibited significantly inferior mechanical characteristics compared to other polymers, with noticeable delamination and a pronounced noise signal observed after the stretching procedure. This poor mechanical behavior renders Gel unsuitable for direct application as a wound dressing (Figure 4b). To overcome this limitation, the mechanical properties of Janus nanofibers were enhanced by incorporating CA, leading to improved mechanical characteristics.

However, the addition of CUR and ZnO NPs in F1 and F2 resulted in a decrease in the mechanical properties compared to F0. This can be attributed to the loose structure of the fiber membrane caused by the presence of CUR and ZnO NPs. The nanoparticles filled the gaps between the fibers, reducing fiber spacing and hindering fiber movement, ultimately leading to a decrease in the overall mechanical properties of the fiber membrane [43,44].

### 3.5. In Vitro Drug Release

**Figure 5:**
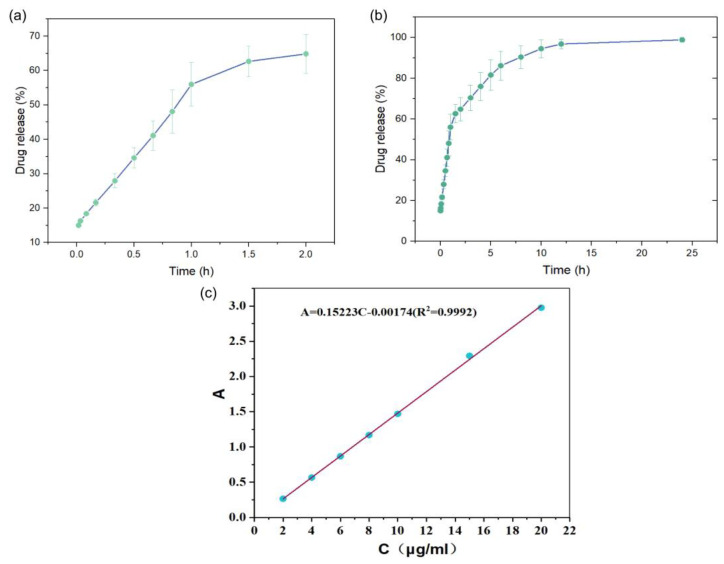
(**a**) Release of CUR from F1 nanofiber over 2 h. (**b**) Release of CUR from F1 nanofiber in 24 h. (**c**) Linear relationship between CUR solution concentration and absorbance.

A linear fit analysis was performed to establish the relationship between the configured CUR standard solution and absorbance. The analysis revealed a linear correlation between the concentration (C) of CUR and absorbance (A), described by the equation A = 0.15223C − 0.00174 (R2 = 0.9992) (Figure 5c). By utilizing this linear relationship, we estimated the concentration of released medication and constructed the release profile of CUR.

The release profile exhibited an initial burst release of CUR from the Janus nanofiber, with more than 60% of CUR released within the first 2 h (Figure 5a). Subsequently, CUR demonstrated a consistent and sustained release pattern, resulting in a total release of 98.85 ± 1.25% after 24 h (Figure 5b). This release behavior of CUR allows for an initial high concentration to effectively inhibit bacterial activity, followed by a sustained release to maintain bacterial inhibition without causing cytotoxicity or interfering with wound healing processes. Polymers are exploited to modify the release behaviors of loaded drug molecules, and both the type of polymer and their formats and structural characteristics have a remarkable influence on the drug release profile [45,46,47,48]. The modified drug delivery systems based on the Janus nanofibers are still very limited but may be rapidly expanded in the near future.

### 3.6. In Vitro Antimicrobial Properties

**Figure 6:**
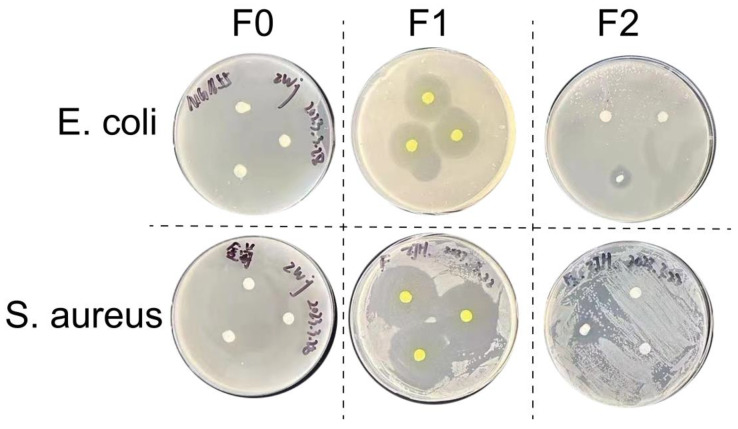
Antibacterial activities of F0, F1, F2 nanofibers against *Escherichia coli* and *Staphylococcus aureus*.

Both ZnO and CUR possess potent antibacterial properties, effectively combating *Staphylococcus aureus* and *Escherichia coli*. However, CUR exhibits superior antibacterial capabilities compared to ZnO. After 24 h of incubation, CUR-loaded nanofibers displayed larger inhibition zones, with a diameter of 2.5 cm for *E. coli* and 3.5 cm for *S. aureus* (Figure 6). On the other hand, ZnO demonstrated weaker antibacterial effects, resulting in smaller inhibition zones.

## 4. Conclusions

The present study successfully developed nanofiber antimicrobial dressings with a Janus structure by utilizing the hydrophilic polymer Gel and the hydrophobic CA. These Janus nanofibers combine high biocompatibility with the ability to regulate drug release. The incorporation of antimicrobial medicines and nanoparticles into the nanofiber allowed for an investigation of their impact on the antibacterial characteristics.

The results demonstrated the effective preparation of Janus nanofibers using a specialized needle in the side-by-side electrospinning process. Extensive optimization was conducted for both types of nanofibers to maximize their respective benefits. The addition of Gel enhanced the hydrophilicity of the nanofibers, resulting in improved biocompatibility of the Janus nanofiber membrane. This enhanced hydrophilicity facilitated cell adhesion and proliferation on the hydrophilic surface. Furthermore, the inclusion of CA addressed the substandard mechanical characteristics of Gel and introduced novel mechanisms for drug release.

The Janus nanofiber membrane, loaded with drugs, exhibited superior antibacterial characteristics, primarily due to the exceptional antimicrobial properties of CUR. This nanofiber wound dressing effectively prevented early-stage wound infections. While ZnO demonstrated weaker antimicrobial properties compared to other substances, the retention of NPs on the nanofibers allowed Janus nanofibers containing ZnO NPs to possess a prolonged antimicrobial effect. Aside from ZnO, other nanoparticles including Au, Ag, and Gu have been investigated as antimicrobial agents. These nanoparticles have been incorporated into nanofibers and have demonstrated effective antibacterial properties [19,20,21,22,23,24,25,26,27,28,29,30,31,32,33,34,35,36,37,38,39,40,41,42,43,44,45,46,47,48,49,50,51]. This prolonged effect is particularly advantageous for the treatment of chronic wounds as it helps prevent recurring infections during the healing process.

In summary, the Janus nanofiber membranes developed in this study, loaded with two distinct antibacterial active compounds, provide a promising solution for addressing both acute and chronic wound issues. The straightforward production procedure holds promise for future clinical applications.

## Figures and Tables

**Table 1:** Electrospun parameters.

Group	Crescent Side (*w*/*v*)	Circular Side (*w*/*v*)	Flow Rate (mL/h)	Voltage (kV)
CA	/	12% CA	1.5	9.0
Gel	/	14% Gel	1.5	8.5
F0	12% CA	14% Gel	1.5:1.5	9.5
F1	12% CA + 2% CUR	14% Gel	1.5:1.5	9.5
F2	12% CA + 2% ZnO	14% Gel	1.5:1.5	9.5

## Data Availability

The data presented in this study are available on request from the corresponding author.
